# Efficacy and safety of lubiprostone combined with polyethylene glycol electrolyte powder for bowel preparation in patients classified by risk level: a randomised trial

**DOI:** 10.3389/fonc.2025.1620794

**Published:** 2025-09-25

**Authors:** Jian Song, Hong-Liang Li, Xu-Fei Qi, Chang-Xi Chen, Yue-Mei Xu

**Affiliations:** Department of Gastroenterology, The Affiliated People’s Hospital of Ningbo University, Ningbo, China

**Keywords:** bowel preparation, colonoscopy, effect, lubiprostone, polyethylene glycol electrolyte powder, safety

## Abstract

**Objective:**

This study aimed to assess the efficacy and safety of lubiprostone combined with polyethylene glycol (PEG) electrolyte powder for bowel preparation in patients classified by risk level.

**Methods:**

The following factors were considered to be associated with inadequate bowel preparation: constipation (meeting Rome IV criteria), BMI > 25 kg/m², history of inadequate bowel preparation, colorectal surgery, diabetes, stroke, or spinal cord injury, Parkinson’s disease, and use of tricyclic antidepressants or anesthetics. A total of 424 patients scheduled for colonoscopy were included and categorized into high-risk and low-risk groups based on bowel preparation risk factors. These patients were then randomly assigned to block groups. The high-risk group was further subdivided into the H-PEG subgroup (PEG, 99 cases) and the H-PEG+L subgroup (PEG + lubiprostone, 105 cases), while the low-risk group was divided into the L-PEG subgroup (PEG, 103 cases) and the L-PEG+L subgroup (PEG + lubiprostone, 102 cases). The following parameters were assessed in each group: time to first bowel movement, total bowel movement count, adverse reactions during preparation, insertion and withdrawal durations, Boston Bowel Preparation Scale (BBPS) scores, rate of adequate bowel preparation, colorectal adenoma detection rates, and willingness to undergo repeated bowel preparation.

**Results:**

During bowel preparation, the H-PEG+L subgroup exhibited a shorter time to first bowel movement and a higher total bowel movement count than the H-PEG subgroup (*p* < 0.05). Similarly, the L-PEG+L subgroup demonstrated a reduced time to first bowel movement and increased total bowel movements compared to the L-PEG subgroup (*p* < 0.05). Within the high-risk group, the H-PEG+L subgroup had higher BBPS scores, a higher rate of adequate bowel preparation (*p* < 0.05), and greater willingness to undergo repeated bowel preparation (*p* < 0.05). No significant differences were found in the low-risk group. Additionally, no differences were observed between groups regarding adverse reactions, insertion duration, withdrawal duration, or adenoma detection rates.

**Conclusion:**

Among patients classified as high risk for inadequate bowel preparation, the combination of lubiprostone and PEG electrolyte powder significantly enhances BBPS scores and bowel cleanliness compared to PEG electrolyte powder alone, without increasing the incidence of adverse events, and is more acceptable for repeated bowel preparation. However, in patients classified as low-risk, the addition of lubiprostone does not provides additional benefits.

**Clinical trial registration:**

https://clinicaltrials.gov, identifier ChiCTR2400088155.

## 1 Introduction

Colorectal cancer ranks as the third leading cause of cancer-related mortality worldwide, with its incidence steadily rising in recent years (1). Data on its global burden highlight that timely screening, early detection, and prompt treatment strategies effectively optimize medical resource allocation (2, 3). Growing evidence suggests a shift toward younger screening ages, with current recommendations advocating screening initiation at age 45 to lower colorectal cancer incidence and mortality (4, 5). Colonoscopy remains the most effective and widely utilized method for colorectal cancer screening (5). The adequacy of bowel preparation is a critical determinant of colonoscopy outcomes and is routinely assessed in clinical practice across endoscopy centers (6). The risk of insufficient bowel preparation varies across populations, making optimal bowel cleansing particularly essential for high-risk groups (7).

Polyethylene glycol electrolyte powder (PEG) is the most widely used bowel cleanser. A high-volume PEG (4L solution) is the most commonly used regimen for bowel preparation prior to colonoscopy, while in China, the 3L solution is recommended more frequently (8). While offering high safety and effective bowel cleansing, its poor palatability and large volume frequently cause adverse effects such as nausea, vomiting, and abdominal distension, leading to preparation intolerance or inadequate bowel preparation (9). More critically, patients with specific conditions such as chronic constipation, obesity, or a history of colorectal surgery experience greater difficulty in attaining high-quality bowel preparation compared to the general population. Consequently, various modified regimens incorporating PEG have been developed, including combinations with ascorbic acid, bisacodyl, and Lubiprostone, among others (9–13).

Lubiprostone, a locally acting chloride channel activator, selectively targets intestinal CIC-2 channels to stimulate fluid secretion and enhance colonic motility, thereby establishing its clinical efficacy in managing diverse constipation-related disorders. Studies demonstrate that a split-dose 2L PEG regimen combined with lubiprostone achieves bowel cleansing efficacy comparable to the standard 4L split-dose PEG (12). However, additional research indicates that adjunctive lubiprostone provides no incremental benefit for bowel preparation adequacy in patients with chronic constipation (13).

Given lubiprostone’s potential to enhance bowel preparation quality, its specific effects in patients with varying bowel preparation risks to achieve more precise individualized treatment were further examined in this study. Currently, no definitive research data are available in this area. Lubiprostone’s effects in patients undergoing colonoscopy were investigated in this study at the Affiliated People’s Hospital of Ningbo University Medical School, Ningbo University.

## 2 Data and methods

### 2.1 Patients

A randomized, single-blind, prospective study was conducted, enrolling patients aged 18 to 70 scheduled for colonoscopy at the Department of Gastroenterology, Affiliated People’s Hospital of Ningbo University Medical School, Ningbo University, between August and December 2024. The trial was registered with www.chictr.org.cn (registration date 13/08/2024, ChiCTR2400088155).

Inclusion criteria for high-risk bowel preparation group: (1) Aged 18 to 70 and scheduled for colonoscopy. (2) Presence of at least one bowel preparation risk factor: chronic constipation (meeting Rome IV criteria), BMI > 25 kg/m², history of inadequate bowel preparation, colorectal surgery, diabetes, stroke, or spinal cord injury; Parkinson’s disease; use of tricyclic antidepressants or anesthetics (14, 15). Inclusion criteria for low-risk bowel preparation group: (1) Aged 18 to 70 and scheduled for colonoscopy. (2) Absence of the bowel preparation risk factors mentioned above. The high-risk and low-risk bowel preparation groups share identical exclusion criteria: (1) History of allergy to Lubiprostone or PEG electrolyte powder. (2) Laxative use within the past 7 days. (3) Severe heart, liver, or kidney disease. (4) History of bowel obstruction, intestinal perforation, electrolyte disturbances, or active inflammatory bowel disease. (5) History of psychiatric disorders, inability to cooperate with the examination, or use of psychiatric medications. (6) Pregnancy or breastfeeding.

All patients in this study were fully informed of the associated risks, and they voluntarily participated, and provided written informed consent. The study was conducted following approval from the Medical Ethics Committee of the Affiliated People’s Hospital of Ningbo University (No: 2024-(Research)-073).

### 2.2 Test design

Patients completed a bowel preparation risk factor screening questionnaire (QUESTIONNAIRE PART S1) via WeChat after confirming study participation, assisted by study staff. Based on the results, they were classified into hig-risk and low-risk bowel preparation groups. In the high-risk bowel preparation group, using a computer-generated random number table, patients were randomly assigned at a 1:1 ratio to one of two subgroups: the H-PEG subgroup and the H-PEG+L subgroup. Similarly, with the same randomization method, the low-risk bowel preparation group was divided into the L-PEG subgroup and the L-PEG+L subgroup. The randomisation was adequately concealed, and the study staff was responsible for its implementation.

A 3L PEG solution was administered for bowel cleansing in the H-PEG and L-PEG subgroups. The procedure involved the following steps: One box of PEG electrolyte powder [brand name: Hengkang Zhengqing, National Drug Approval No: H20020031, Jiangxi Hengkang Pharmaceutical Co., Ltd., Compound Polyethylene Glycol Electrolyte Powder (I)], containing three packets (A, B, and C), was consumed the night before the examination. Packet A contained 0.74g of potassium chloride and 1.68g of sodium bicarbonate; packet B contained 1.46g of sodium chloride and 5.68g of sodium sulfate; packet C contained 60g of PEG 4000. Two additional boxes of PEG electrolyte powder (2L PEG) were taken 4 to 6 hours before the examination, with adjustments based on the appointment time. In addition, one capsule of placebo, which was similar in shape and smell to lubiprostone, was administered at 2:00 PM on the afternoon prior to the colonoscopy.

Lubiprostone was combined with a 3L PEG electrolyte powder solution for bowel cleansing in the H-PEG+L and L-PEG+L subgroups. One capsule of Lubiprostone soft gel (brand name: Changfan; Nanjing Chia Tai Tianging Pharmaceutical Co., Ltd.; National Drug Approval No: H20233802; strength: 24 μg per capsule) was administered at 2:00 PM on the afternoon before the colonoscopy. A 1L PEG solution was consumed that evening, followed by a 2L PEG solution taken 4 to 6 hours before the examination. The PEG solution was completely consistent with that in the H-PEG and L-PEG subgroups.

All patients undergoing bowel preparation were instructed to take 30 mL of simethicone emulsion (brand name: Espumisan; Berlin-Chemie AG; National Drug Approval No: HJ20160184; strength: 30 mL per bottle) within 30 to 60 minutes after the final dose of the laxative.

Before bowel preparation, all patients received a printed information sheet outlining dietary precautions and the bowel preparation protocol. They also had access to an online video explanation. Additionally, endoscopy department nurses provided specialized education to ensure that patients fully understood the process. Two days before the colonoscopy, patients adhered to a low-residue, low-fiber diet, and on the day before the procedure, they consumed only clear liquids. On the examination day, scheduled between 1:30 PM and 5:00 PM, patients were required to fast. Prior to colonoscopy at the endoscopy center, patients underwent an adherence inquiry of their bowel preparation process. Those failing to meet the predetermined protocol requirements were excluded from the trial.

### 2.3 Data collection

After confirming participation in the study, patients immediately completed a questionnaire (QUESTIONNAIRE PART S1) collecting data on age, gender, BMI, chronic constipation, diabetes, history of inadequate bowel preparation, colorectal surgery, and other bowel preparation risk factors. Before colonoscopy, patients completed another questionnaire (QUESTIONNAIRE PART S2) assessing their bowel preparation experience, including time to first bowel movement, total bowel movements, occurrence of adverse reactions (e.g., nausea, vomiting, abdominal pain, bloating, dizziness, headache, fatigue), and willingness to repeat bowel preparation.

Bowel preparation cleanliness was assessed using the Boston Bowel Preparation Scale (BBPS), a reliable and widely used tool (16). Before the study, colonoscopy physicians received training on the scoring scale, with standard reference images provided for comparison. The colon is divided into three segments: the right colon (ileocecal region and ascending colon), the transverse colon (hepatic flexure, transverse colon, splenic flexure), and the left colon (descending colon, sigmoid colon, rectum). Scoring criteria: 0 points—solid stool prevents visualization of the colonic mucosa; 1 point—most of the enteric cavity is obscured by fecal residue, coloring, or opaque fluid, with limited mucosal visibility; 2 points—minor obstruction from residue, coloring, or opaque fluid, but most of the mucosa is visible; 3 points—entire mucosa is well-prepared with no residue or opaque fluid. The total score (0-9) is the sum of the three segments, with higher scores indicating better bowel cleanliness. Adequate bowel preparation is defined as a total score of ≥6 and ≥2 per segment (17).

The detection rate of colorectal adenomas was determined by dividing the number of patients who had at least one adenoma detected (confirmed through biopsy or complete removal with pathological examination) during colonoscopy by the total patients in each subgroup. All enrolled patients underwent random assignment to three senior endoscopists (each having performed over 3,000 colonoscopies, physician IDs: 10488, 10671, 11515) for their procedures, with withdrawal times maintained at ≥ 6 minutes. Endoscopists were blinded to both bowel preparation risk stratification and study medication assignments.

### 2.4 Statistical methods

The sample size for this study was determined by applying a formula suitable for research concerning multiple independent proportions. The proportion of adequate bowel preparation achieved with PEG alone generally ranges from 56% to 76%. Conversely, the addition of lubiprostone to PEG has been demonstrated to improve the quality of bowel preparation to 90.3% (18). Based on these proportions, the sample size was calculated to ensure adequate statistical power. A sample size of at least 41 subjects was required to attain 80% power with a two - sided α of 0.05.

Measurement data were presented as mean ± standard deviation (x ± s). For intergroup comparisons, independent sample t-tests were applied to normally distributed data, while non-normal data were analyzed using non-parametric tests (Mann-Whitney U test). Categorical variables were expressed as rates, with intergroup comparisons conducted via the χ² test. All statistical analyses were performed using SPSS Version 28.0 (IBM SPSS Statistics, USA), and statistical significance was set at *p* < 0.05.

## 3 Results

**Figure 1:**
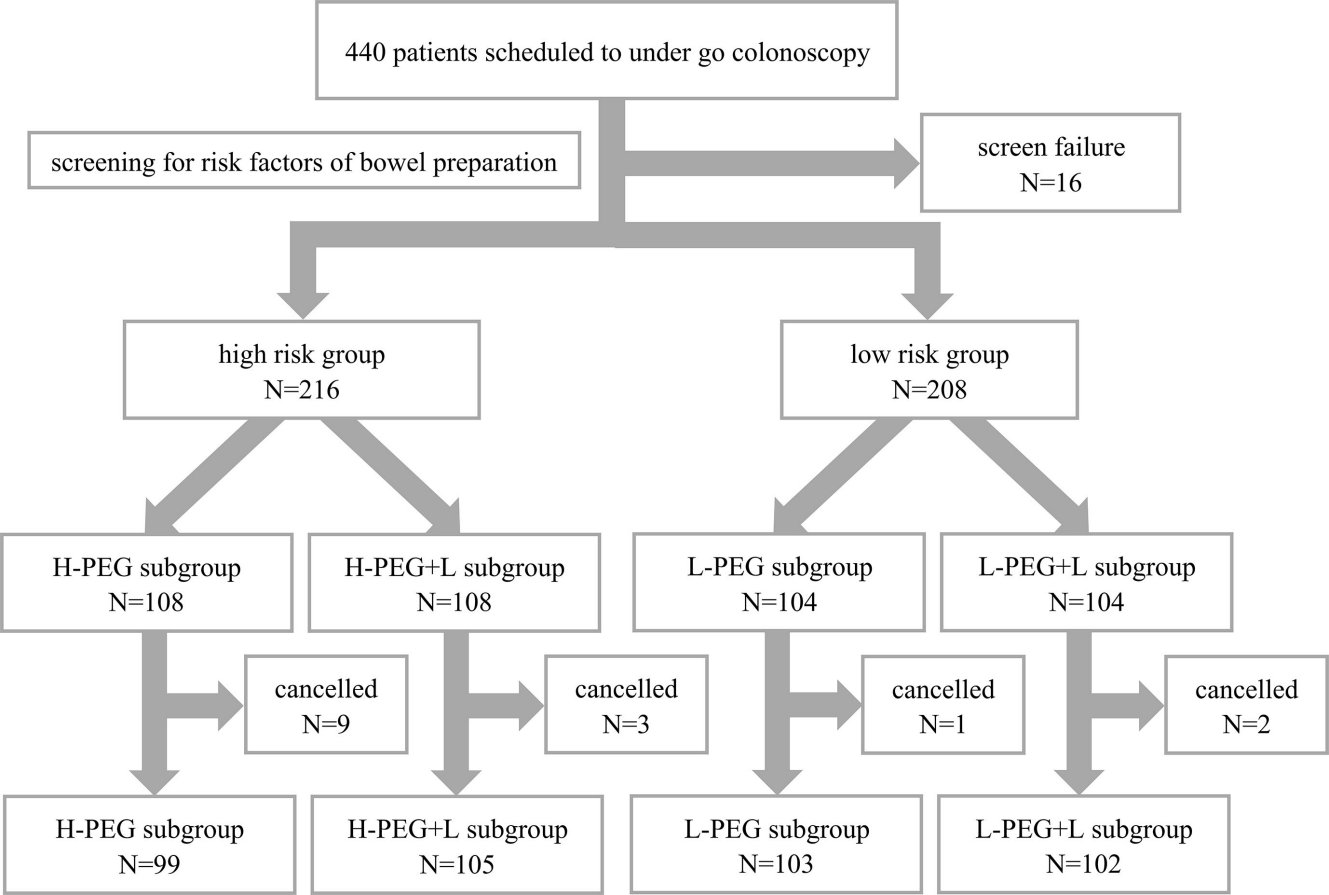
Study flowchart.

A total of 440 patients were recruited for this study. After excluding 16 patients who met the exclusion criteria, 424 patients remained eligible, including 216 in the high-risk bowel preparation group and 208 in the low-risk group. Random number allocation assigned patients at high risk to either the H-PEG or H-PEG+L subgroup, while patients at low risk were assigned to either the L-PEG or L-PEG+L subgroup. Before undergoing colonoscopy, fifteen patients withdrew from the study (ten temporarily canceled the examination, and five did not adhere to the prescribed medication regimen). Ultimately, 409 patients were included in the final analysis: 99 in the H-PEG subgroup, 105 in the H-PEG+L subgroup, 103 in the L-PEG subgroup, and 102 in the L-PEG+L subgroup, as presented in **Figure 1**. These patients all successfully completed the colonoscopy (no missing data). All colonoscopies had been performed under anesthesia, with propofol utilized for the induction and maintenance of general anesthesia.

### 3.1 Basic analysis of characteristics of each group

The high-risk bowel preparation group was categorized into the H-PEG and H-PEG+L subgroups based on the intervention measures. No statistically significant differences were observed between these subgroups regarding age, gender, BMI, chronic constipation, diabetes, history of inadequate bowel preparation, or history of abdominal surgery (*p* > 0.05). In the low-risk group, due to different inclusion criteria, comparisons between the L-PEG and L-PEG+L subgroups were limited to age, gender, and BMI. No statistically significant differences were found between these subgroups (*p* > 0.05) (**Table 1**).

**Table 1:** Basic characteristics analysis.

Characteristics	High-risk			Low-risk		
	H-PEG (N=99)	H-PEG+L (N=105)	P	L-PEG (N=103)	L-PEG+L (N=102)	P
Age (years)	52.6 ± 11.5	53 ± 11.6	0.739	53.3 ± 11.1	52.5 ± 12.7	0.859
Female, n (%)	54(54.55%)	50(47.62%)	0.323	56(54.37%)	45(44.12%)	0.142
BMI	26.39 ± 3.64	25.99 ± 3.21	0.209	21.36 ± 1.98	21.9 ± 1.89	0.596
Chronic constipation, n (%)	13(13.13%)	18(17.14%)	0.425	/	/	/
Diabetes, n (%)	19(19.19%)	20(19.05%)	0.979			
History of inadequate bowel preparation, n (%)	12(12.12%)	15(14.29%)	0.648	/	/	/
History of abdominal surgery, n (%)	11(11.11%)	13(12.38%)	0.778	/	/	/

BMI, Body Mass Index.

### 3.2 Comparison of bowel movement and adverse reaction rates in each group

The H-PEG+L subgroup demonstrated a shorter interval to the first bowel movement and a greater total number of bowel movements than the H-PEG subgroup in the high-risk bowel preparation group, with statistically significant differences (*p* < 0.05). Similarly, in the low-risk bowel preparation group, the L-PEG+L subgroup exhibited a shorter interval to the first bowel movement and a higher total number of bowel movements compared to the L-PEG subgroup, with statistically significant differences (*p* < 0.05) (**Table 2**).

**Table 2:** Comparison of bowel movement and adverse reaction rates.

Characteristics	High-risk			Low-risk		
	H-PEG (N=99)	H-PEG+L (N=105)	P	L-PEG (N=103)	L-PEG+L (N=102)	P
Interval to the first bowel movement (hour)	1.75 ± 0.74	1.34 ± 0.64	0.000^*^	1.55 ± 0.76	1.32 ± 0.59	0.026^**^
Total count of bowel movement (hour)	11.0 ± 3.6	12.6 ± 3.5	0.003^*^	12.5 ± 3.5	14.3 ± 3.5	0.001^**^
Nausea, n (%)	40(40.40%)	37(35.24%)	0.447	36(34.95%)	46(45.10%)	0.138
Vomiting, n (%)	12(12.12%)	8(7.62%)	0.280	10(9.71%)	13(12.75%)	0.491
Abdominal pain, n (%)	8(8.08%)	5(4.76%)	0.332	4(3.88%)	2(1.96%)	0.688
Abdominal bloating, n (%)	25(25.25%)	22(20.95%)	0.466	15(14.56%)	10(9.80%)	0.298
Dizziness, n (%)	5(5.05%)	7(6.67%)	0.624	5(4.85%)	6(5.88%)	0.744
Headache, n (%)	2(2.02%)	2(1.90%)	0.953	1(0.97%)	1(0.98%)	0.995
Fatigue, n (%)	8(8.08%)	9(8.57%)	0.899	8(7.77%)	6(5.88%)	0.593
Tiredness, n (%)	5(5.05%)	5(4.76%)	0.924	4(3.88%)	3(2.94%)	0.710

*H-PEG vs H-PEG+L comparison, *p* < 0.05; **L-PEG vs L-PEG+L comparison, *p* < 0.05.

No significant statistical differences were observed between the two subgroups in the high-risk group (H-PEG vs. H-PEG+L) or the two subgroups in the low-risk group (L-PEG vs. L-PEG+L) regarding nausea, vomiting, abdominal pain, bloating, dizziness, headache, fatigue, and tiredness (*p* > 0.05) (**Table 2**).

### 3.3 Comparison of insertion and withdrawal times in each group

No significant statistical differences were observed in insertion time or withdrawal time between the subgroups in both the high-risk bowel preparation group (H-PEG vs. H-PEG+L) and the low-risk bowel preparation group (L-PEG vs. L-PEG+L) (*p* > 0.05) (**Table 3**).

**Table 3:** Comparison of insertion and withdrawal time.

Characteristics	High-risk			Low-risk		
	H-PEG (N=99)	H-PEG+L (N=105)	P	L-PEG (N=103)	L-PEG+L (N=102)	P
Insertion time (min)	6.27 ± 3.56	7.14 ± 4.28	0.170	6.76 ± 4.13	6.35 ± 3.35	0.330
Withdrawal time (min)	7.55 ± 1.17	7.69 ± 1.31	0.587	7.85 ± 1.53	7.7 ± 1.33	0.615

### 3.4 Comparison of BBPS scores and rate of adequate bowel preparation in each group

The H-PEG+L subgroup of the high-risk bowel preparation group demonstrated higher BBPS scores for each bowel segment and a greater total score than the H-PEG subgroup, with statistically significant differences (*p* < 0.05). Similarly, the rate of adequate bowel preparation was also higher in the H-PEG+L subgroup (*p* < 0.05). Conversely, in the low-risk bowel preparation group, no significant differences in BBPS scores and the rate of adequate bowel preparation were observed between the L-PEG and L-PEG+L subgroups (*p* > 0.05) (**Table 4**).

**Table 4:** Comparison of BBPS scores and rate of adequate bowel preparation.

Characteristics	High-risk			Low-risk		
	H-PEG (N=99)	H-PEG+L (N=105)	P	L-PEG (N=103)	L-PEG+L (N=102)	P
Right colon	1.45 ± 0.5	1.88 ± 0.49	0.000^*^	1.79 ± 0.52	1.9 ± 0.41	0.064
Transverse colon	1.81 ± 0.7	2.15 ± 0.62	0.000^*^	2.15 ± 0.69	2.27 ± 0.51	0.229
Left colon	1.99 ± 0.79	2.32 ± 0.67	0.002^*^	2.28 ± 0.69	2.43 ± 0.55	0.170
Total score	5.25 ± 1.83	6.35 ± 1.61	0.000^*^	6.21 ± 1.74	6.74 ± 1.28	0.056
Rate of adequate bowel preparation	45(45.45%)	85(80.95%)	0.000^*^	76(73.79%)	87(85.29%)	0.041

*H-PEG vs H-PEG+L comparison, *p* < 0.05.

BBPS, Boston Bowel Preparation Scale.

In addition, data were analysed for the H-PEG subgroups and L-PEG subgroups, as well as the H-PEG+L subgroups and L-PEG+L subgroups. Within the PEG group, the H-PEG subgroup had lower BBPS scores for each bowel segment and a lower total score than the L-PEG subgroup, with statistically significant differences (*p < 0.05*). The rate of adequate bowel preparation was the same in the two groups (*p < 0.05*). However, there were no significant differences in BBPS scores or the rate of adequate bowel preparation between the H-PEG+L subgroup and the L-PEG+L subgroup (*p* > 0.05) (**Supplementary Table S1**).

### 3.5 Comparison of colorectal adenoma detection rates in each group

No significant statistical differences were observed in colorectal adenoma detection rates (*p* > 0.05) in both the high-risk bowel preparation subgroups (H-PEG vs. H-PEG+L) and the low-risk bowel preparation subgroups (L-PEG vs. L-PEG+L) (**Table 5**).

**Table 5:** Comparison of colorectal adenoma detection rate and willingness to repeat bowel preparation.

Characteristics	High-risk			Low-risk		
	H-PEG (N=99)	H-PEG+L (N=105)	P	L-PEG (N=103)	L-PEG+L (N=102)	P
Colorectal adenoma detection rate	18(18.18%)	31(29.52%)	0.058	29(28.16%)	31(30.39%)	0.725
Willingness for repeat bowel preparation	20(20.20%)	35(33.33%)	0.035^*^	26(25.24%)	28(27.45%)	0.720

*H-PEG vs H-PEG+L comparison, *p* < 0.05.

Furthermore, statistical analyses were conducted for patients aged over 50. In the H-PEG subgroup of patients over 50, there were a total of 61 patients, and the adenoma detection rate was 22.95% (14/61). In the H-PEG+L subgroup of patients over 50, there were 62 patients, with an adenoma detection rate of 32.26% (20/62). Through statistical testing, *p* = 0.249 > 0.05, indicating that there was no significant statistical difference between these two subgroups. Similarly, in the L-PEG subgroup of patients over 50, there were 65 patients, and the adenoma detection rate was 33.85% (22/65). In the L-PEG+L subgroup of patients over 50, there were 63 patients, and the adenoma detection rate was 36.51% (23/63). After statistical testing, P = 0.753 > 0.05, suggesting that there was also no significant statistical difference between these two subgroups.

### 3.6 Comparison of willingness to repeat bowel preparation in each group

Willingness to repeat bowel preparation was higher in the H-PEG+L subgroup than in the H-PEG subgroup, with a statistically significant difference (*p* < 0.05) in the high-risk bowel preparation group. However, in the low-risk bowel preparation group, no significant statistical difference was observed between the L-PEG and L-PEG+L subgroups (*p* > 0.05) (**Table 5**).

## 4 Discussion

Colonoscopy is recognized as the gold standard for colorectal cancer screening, offering both diagnostic and therapeutic capabilities (19). For patients undergoing colonoscopy, bowel preparation remains a major challenge and is often perceived as the most burdensome aspect of the procedure (11, 20).

Attaining satisfactory bowel cleanliness is more challenging in high-risk populations with specific bowel preparation risk factors. This difficulty may result in a lower detection rate of polyps and adenomas, screening failures, and a lower likelihood of repeated procedures (21). Therefore, exploring additional bowel preparation strategies beyond standard protocols is essential.

Lubiprostone is an FDA-approved medication for treating idiopathic constipation. It functions as a locally acting type 2 chloride ion channel (ClC-2) agonist. By activating ClC-2 channels on the apical surface of intestinal epithelial cells, lubiprostone enhances intestinal fluid secretion and increases bowel motility, facilitating defecation. It does not lead to severe complications, and the most common side effects are mild nausea, diarrhea, and abdominal pain (22, 23).

Recent research has indicated that lubiprostone acts as a non-selective cAMP-gated ion channel activator. By stimulating the E-type prostanoid receptor 4 (EP4 receptor), it elevates intracellular cAMP levels, subsequently activating multiple cAMP-gated ion channels, including Cystic Fibrosis Transmembrane Conductance Regulator (CFTR) and ClC-2. The secretory effects of lubiprostone on intestinal epithelial cells primarily involve CFTR, with ClC-2 playing a secondary role (24). Additionally, some studies suggest that lubiprostone indirectly activates the TRPC4 channel via the EP3 receptor, enhancing distal colon contraction strength (25).

Although lubiprostone is not a standard medication for bowel preparation before colonoscopy, several studies have assessed its potential effects. Grigg et al. conducted a single-blind, randomized trial involving 60 diabetic patients to compare the effects of PEG combined with lubiprostone versus PEG alone. However, the study was prematurely terminated due to funding limitations, leading to a small sample size. Although the statistical difference was not significant, a trend toward improved bowel preparation was observed. Grigg et al. suggested that if both study groups had completed the study as planned, a statistically significant difference might have been achieved (26).

In a randomized controlled trial, Sirinawasatien et al. included 140 patients to compare the bowel cleansing effects of a 2L PEG combined with 24 µg of lubiprostone regimen versus a 4L PEG regimen. The findings demonstrated that the 2L PEG combined with lubiprostone regimen achieved bowel cleanliness (based on BBPS scores) comparable to the 4L PEG regimen, with no additional adverse events and a reduced PEG volume (12).

The effect of PEG combined with lubiprostone on colonoscopy was examined by Banerjee et al. in a randomized, double-blind, placebo-controlled trial involving 442 patients. Compared to PEG alone, lubiprostone administered before PEG significantly improved the quality of bowel preparation for colonoscopy (8).

Given the potential role of lubiprostone in enhancing bowel preparation quality, this study conducted a single-center randomized controlled trial to evaluate the efficacy and safety of a lubiprostone combined with PEG regimen compared to the traditional PEG regimen in patients classified by risk level. The objective was to provide a clinical reference for bowel preparation across different risk groups.

The experimental data indicated that lubiprostone use significantly shortened the interval to the first bowel movement and increased the total number of bowel movements in both high-risk and low-risk groups. This aligns with lubiprostone’s known effect of increasing intestinal fluid volume and promoting bowel motility. Additionally, no significant differences in adverse events were observed between the lubiprostone subgroup and the control group, suggesting its safety for bowel preparation.

Subgroup comparisons across risk groups revealed no significant differences in insertion or withdrawal time. These findings indicated that lubiprostone neither adversely affected the procedure nor offered additional benefits.

Bowel cleanliness significantly improved in the lubiprostone-PEG subgroup of high-risk group, compared to the control, with differences observed in the right, transverse, and left colon, as well as in total scores. However, no significant improvement was noted in the low-risk group, suggesting that lubiprostone provides greater benefits for patients at high risk but offers no additional advantages for low-risk individuals. At the same time, we compared the bowel preparation BBBPS between the H-PEG and L-PEG subgroups. This confirmed that bowel preparation is more challenging for high-risk groups than for low-risk groups.

Although bowel cleanliness improved with lubiprostone in patients at high risk, the colorectal adenoma detection rate did not significantly increase, despite a trend toward improvement. We added the colorectal adenoma detection rate for patients aged over 50. Unfortunately, we still did not find a statistically significant difference. A larger sample size might yield a statistically significant difference. This study required colonoscopy-performing physicians to maintain a minimum withdrawal time of 6 minutes, as recommended in clinical practice to enhance adenoma detection rates (27, 28). Recent evidence indicates that extending withdrawal time beyond 9 minutes may further improve detection rates (29). Adequate withdrawal time enhances adenoma detection and compensates for missed diagnoses due to suboptimal bowel preparation. This could explain why some patients with inadequate bowel preparation still had adenomas detected when examination time was extended (30). Consequently, this factor may have contributed to the lack of a significant difference in adenoma detection rates in this study.

A higher acceptance rate for repeat bowel preparation was observed among patients at high risk who used lubiprostone in combination. This may be attributed to more efficient bowel movements in this subgroup, leading to better bowel cleanliness and reduced anxiety during preparation. However, no similar effect was observed in patients with low risk. Additional interventions can assist patients in achieving optimal bowel cleanliness through various methods, thereby alleviating anxiety. This finding aligns with the study results of Wen et al. (31).

In this study, patients with inadequate bowel preparation were recommended to undergo a repeat colonoscopy within one year. The selected bowel preparation regimen should be a high-volume PEG regimen combined with lubiprostone or another regimen proven by evidence-based medicine to effectively enhance bowel preparation. These patients were kept under continuous observation, and the results were recorded.

The overall quality of bowel preparation among the patients participating in the study was not high. This was related to the fact that the mainstream bowel preparation method in China is using 3L of PEG solution. There was a compromise on the large - volume PEG, and making improvements based on the 3L PEG solution was a good exploration and choice.

This study has several limitations. First, as a single-center study, it lacks supporting data from other centers using the same design. Second, the optimal dosage, timing, and duration of lubiprostone administration remain under investigation, as its regimen is still in the exploratory phase. Third, the sample size is insufficient, and larger future studies will strengthen the evidence, further enhancing the reliability and validity of the findings. Finally, this study differentiated between populations with different risks for bowel preparation. With a larger sample size, it might have been possible to make a more detailed delineation of the different elements of the risk factors, which would have made the results more precise.

## 5 Conclusion

The combination of lubiprostone and PEG electrolyte powder significantly improved BBPS scores compared to PEG electrolyte powder alone in patients at high risk. This regimen enhanced bowel cleanliness without increasing adverse reactions, and patients exhibited a higher willingness to repeat bowel preparation. However, in patients at low risk, lubiprostone offered no additional benefits.

## Data Availability

The original contributions presented in the study are included in the article/**Supplementary Material**. Further inquiries can be directed to the corresponding author.
