# Supplementary material for: Efficacy and safety of lubiprostone combined with polyethylene glycol electrolyte powder for bowel preparation in patients classified by risk level: a randomised trial
Source: Front Oncol. 2025 Sep 25;15:1620794. doi: 10.3389/fonc.2025.1620794 (PMC12507641; doi:10.3389/fonc.2025.1620794)
Supplement: Supplementary file 1 [file DataSheet1.docx]

**QUESTIONNAIRE PART 1**

**Name**：________

**Subject ID**：________

**Sex：** ◻ Male ◻ Female

**Date of Birth**(YYYY-MM-DD)：________

☐ Age 18–70 years → Proceed to screening

☐ Age <18 or >70 years → Screen failure

**Risk Factor Screening:**

1. BMI (kg/m²) >25:  ☐ Yes ☐ No
   *(Calculation: Weight in kg / (Height in m)²)*
2. Chronic constipation (Rome IV Criteria) *:  ☐ Yes ☐ No
3. Diabetes:  ☐ Yes ☐ No
4. History of inadequate bowel preparation:  ☐ Yes ☐ No
5. History of colorectal surgery:  ☐ Yes ☐ No
6. History of stroke/spinal cord injury or Parkinson's disease:  ☐ Yes ☐ No
7. Use of tricyclic antidepressants or analgesics:  ☐ Yes ☐ No

Subjects meeting ANY of the above risk factors will be assigned to the high-risk bowel preparation group.

Subjects with NONE of the above risk factors will be assigned to the low-risk bowel preparation group.

**Exclusion Criteria:**

1. History of allergy to lubiprostone or polyethylene glycol electrolyte powder
2. Laxative use within the past 7 days
3. Concurrent clinically significant cardiac, hepatic, or renal disease
4. Current diagnosis of:
   - Intestinal obstruction/perforation
   - Electrolyte imbalance
   - Active-phase inflammatory bowel disease
5. History of psychiatric disorders, inability to cooperate with the examination, or use of psychiatric medications
6. Pregnancy or breastfeeding

Subjects meeting ANY of Criteria 1-6 will be excluded.

Investigator: ___________

Date: ___________

***Supplementary Note**: The investigator will assist the subject in determining whether there is chronic constipation (Rome IV). The following are the criteria for judgment.

| Onset of constipation symptoms at least 6 months before diagnosis  Below criteria met for the past 3 months |
| --- |
| I. Two or more of the following criteria must be present |
| a. Straining with >25% of defecations |
| b. Lumpy or hard stools with >25% of defecations |
| i. Bristol stool form types 1 and 2 |
| c. Sensation of incomplete evacuation with >25% of defecations |
| d. Sensation of anorectal obstruction/blockage with >25% of defecations |
| e. Manual maneuvers required with >25% of defecations |
| i. Eg, digital evacuations, support for the pelvic floor |
| f. Fewer than 3 spontaneous defecations per week |
| II. Loose stools are rare without administration of laxatives |
| III. Insufficient criteria for irritable bowel syndrome |
