# Supplementary material for: Efficacy and safety of lubiprostone combined with polyethylene glycol electrolyte powder for bowel preparation in patients classified by risk level: a randomised trial
Source: Front Oncol. 2025 Sep 25;15:1620794. doi: 10.3389/fonc.2025.1620794 (PMC12507641; doi:10.3389/fonc.2025.1620794)
Supplement: Supplementary file 2 [file DataSheet2.docx]

**QUESTIONNAIRE PART 2**

***(To be completed within 2 hours before colonoscopy)***

**Name**：________

**Subject ID**：________

**Preparation Date:** ________

**A. EFFICACY METRICS**

1. Time to first bowel movement: ______ hours
   *(From start of prep to first bowel movement)*
2. Total bowel movements: ______
   *(During active preparation period)*

**B. ADVERSE EVENTS MONITORING**

| (Check if present during preparation) |
| --- |

| Symptom | |  | Symptom | |
| --- | --- | --- | --- | --- |
| Nausea | ☐ Yes ☐ No |  | Vomiting | ☐ Yes ☐ No |
| Abdominal pain | ☐ Yes ☐ No |  | Abdominal bloating | ☐ Yes ☐ No |
| Dizziness | ☐ Yes ☐ No |  | Headache | ☐ Yes ☐ No |
| Fatigue | ☐ Yes ☐ No |  | Tiredness | ☐ Yes ☐ No |

**C. PATIENT ACCEPTABILITY**

Willingness to repeat preparation: ☐ Yes ☐ No

If "No", specify reason: ___________________________

Investigator: ___________

Date: ___________
