# Supplementary material for: Efficacy and safety of lubiprostone combined with polyethylene glycol electrolyte powder for bowel preparation in patients classified by risk level: a randomised trial
Source: Front Oncol. 2025 Sep 25;15:1620794. doi: 10.3389/fonc.2025.1620794 (PMC12507641; doi:10.3389/fonc.2025.1620794)
Supplement: Supplementary file 3 [file Table1.docx]

Table S1. Comparison of BBPS scores for PEG and PEG+L groups

|  | PEG | | | PEG+L | | |  |
| --- | --- | --- | --- | --- | --- | --- | --- |
|  | H-PEG  (N=99) | L-PEG  (N=103) | P | H-PEG+L  (N=105) | L-PEG+L  (N=102) | P | |
| Right colon | 1.45 ± 0.5 | 1.79 ± 0.52 | .000 | 1.88 ± 0.49 | 1.9 ± 0.41 | .635 | |
| Transverse colon | 1.81 ± 0.7 | 2.15 ± 0.69 | .001 | 2.15 ± 0.62 | 2.27 ± 0.51 | .175 | |
| Left colon | 1.99 ± 0.79 | 2.28 ± 0.69 | .008 | 2.32 ± 0.67 | 2.43 ± 0.55 | .346 | |
| Total score | 5.25 ± 1.83 | 6.21 ± 1.74 | .000 | 6.35 ± 1.61 | 6.74 ± 1.28 | .172 | |
